# Supplementary material for: Optimal cross selection for long-term genetic gain in two-part programs with rapid recurrent genomic selection
Source: Theor Appl Genet. 2018 Jun 6;131(9):1953–66. doi: 10.1007/s00122-018-3125-3 (PMC6096640; doi:10.1007/s00122-018-3125-3)
Supplement: Supplementary file 1 — Supplementary material 1 (PDF 98 kb) [file 122_2018_3125_MOESM1_ESM.pdf]

Optimal cross selection for long-term genetic gain in a two-part genomic selection strategy

Supplementary Material S1:
Additional Material and Methods

Gregor Gorjanc*, R. Chris Gaynor, John M. Hickey

G. Gorjanc, R.C. Gaynor, and J.M. Hickey The Roslin Institute and Royal (Dick) School of Veterinary Studies, University of Edinburgh, Easter Bush Research Centre, Midlothian EH25 9RG, UK

*Corresponding author (gregor.gorjanc@roslin.ed.ac.uk)

Simulation setup 2

Conventional strategy with phenotypic selection 3

Conventional strategy with genomic selection 5

Two-part strategy with genomic selection 6

A cycle of population improvement with truncation selection 6

A cycle of product development 7

Interactions between the two components 7

Costs 7

Optimal cross selection 8

Number of recurrent selection cycles per year 8

Lifting cost constraints 8

References 9

Simulation setup

We initiated simulation by defining a genome with 21 chromosome pairs. Each chromosome had genetic length of 1.43 Morgans and a physical length of $8x10^8$ base pairs. For each chromosome we generated whole chromosome sequences with the Markovian Coalescent Simulator (Chen et al. 2009). In the simulator we set: i) recombination rate to $1.8x10^{-9}$ per base pair (= 1.43 Morgans / $8x10^8$ base pairs), ii) mutation rate to $2x10^{-9}$ per base pair, and iii) effective population size to 50, with linear piecewise increases to 1,000 at 100 generations ago, 6,000 at 1000 generations ago, 12,000 at 10,000 generations ago, and 32,000 at 100,000 generations ago. These values were chosen to roughly follow the evolution of effective population size in wheat (Thuillet et al. 2005; Peng et al. 2011). Finally, we randomly sampled the simulated chromosomes to establish 50 inbred founder genomes.

Out of all segregating variants in founders' sequences we randomly selected 1,000 marker loci per chromosome and 1,000 causal loci per chromosome. Both types of loci were biallelic. In total there were 21,000 markers and 21,000 causal loci. Each causal locus was assigned an additive effect from a normal distribution with a mean of zero and a variance of one divided by the total number of causal loci. The sum of an individual's causal loci effects represents its genetic merit for a polygenic trait. To simulate individual's phenotype we added random error to its genetic merit. Errors were sampled from a normal distribution with mean zero and variance that varied with stages of a breeding program. Specifically, we varied error variance to obtain phenotypes with targeted narrow-sense heritability relative to genetic variance among the founders.

Conventional program with phenotypic selection

The conventional program (Conv) mimicked a wheat breeding program that uses doubled-haploid technology. In Fig. 1 this program is represented under the product development pane. We assumed the use of doubled-haploid technology to enable fair comparison (in terms of cycle time) with the two-part program. Selection was based on phenotypes, either directly on trial performance or indirectly on correlated traits. The key steps of this strategy were:

Year 1 Cross 50 parental lines to produce 100 bi-parental populations. The crosses are sampled without replacement from all possible parent combinations.

Year 1/2 Produce 100 doubled-haploid lines per bi-parental population

Year 3 Plant the 10,000 doubled-haploid lines in headrows. Visually select the best 1,000 lines based on a phenotype with heritability 0.1 (i.e., visual selection).

Year 4 Evaluate the 1,000 lines in a preliminary trial. Select the best 100 lines based on a phenotype with heritability 0.2 (i.e., unreplicated trial). Advance the best 20 lines to next year's crossing block.

Year 5 Evaluate the 100 lines in an advanced trial. Select the best 10 lines based on a phenotype with heritability 0.4 (i.e., small multi-location replicated trial). The 10 lines go to elite trials and next year's crossing block.

Year 6/7 Evaluate the 10 lines in an elite trial. Select the best line based on a phenotype with heritability 0.6 (i.e., large multi-location replicated trial).

Year 8 Release variety.

We used the conventional program with phenotypic selection as a benchmark and designed other programs that had approximately equal costs. As in Gaynor et al. (2017) we assumed that the two dominating costs are creation of double-haploids and genotyping, which were respectively costed at $35 and $20. The conventional program

with phenotypic selection with 10,000 doubled-haploid lines had a per year cost proportional to $350,000 (Table S1.1).

Table S1.1: Breeding program characteristics (number of crosses, number of doubled-haploid lines per cross, total number of doubled-haploid lines, and total cost)

Program	#Crosses	#Lines / cross	#Lines	Cost ($)
Conv	100	100	10,000	350,000
ConvP	100	95	9,500	352,500
ConvH	100	64	6,400	352,000
TwoPart	/	/	6,100	348,300
product development	100	41	4,100	225,500
population improvement	64	31.2	2,000	122,800

[1]Conv – conventional program with phenotypic selection; ConvP/H – conventional program with genomic selection at the preliminary trial stage / headrow stage; TwoPart – two-part program with genomic selection

Conventional program with genomic selection

The conventional program with genomic selection followed closely the conventional program with phenotypic selection. The difference was high-density genotyping and genomic selection of lines for trials and next year's crossing block to reduce cycle time (Fig. 1). We performed genomic selection either at the preliminary trial stage (ConvP) or at the headrow stage (ConvH). This reduced cycle time for one year with genomic selection at the preliminary trial stage or for two years with genomic selection at the headrow stage. The evaluation of lines in ConvP was based both on genomic and phenotypic data, while the headrow evaluation was based solely on genomic data. Genomic selection increases the total costs in comparison to phenotypic selection due to high-density genotyping. We equalized the costs by decreasing the number of doubled-haploid lines per bi-parental population (Table S1.1); to 95 with genomic selection at the preliminary trial stage (9,500 headrows in total, 1,000 of them genotyped at the preliminary trial stage) and to 64 with genomic selection at the headrow stage (6,400 headrows in total, all of them genotyped). The large difference in number of doubled-haploid lines per bi-parental population was needed due to genotyping 1,000 lines at the preliminary trial stage and 6,400 lines at the headrow stage.

Two-part program with genomic selection

The two-part program (TwoPart) differed from the conventional program in explicit separation of product development and population improvement (Fig. 1). The population improvement component is based in a greenhouse that enables several cycles of recurrent genomic selection per year, while the product development component is the same as the conventional program with minor modifications (Table S1.1). We initialized the population improvement component in the last year of burn-in with a half-diallel cross among the existing parents and another round of random crossing to avoid large founder effects and to increase number of recombinations. After the initialization the two components were ready for the year 21. We have run the two-part program under different scenarios: i) truncation selection with two numbers of parents or optimal cross selection in the population improvement component, ii) 1, 2, 3, 4, 5, or 6 cycles of recurrent genomic selection per year, and iii) constrained or unconstrained costs incurred by high-density genotyping.

In the following we first describe: i) a cycle of population improvement with truncation selection, ii) a cycle of product development, iii) interactions between the two components, and iv) how we equalized the costs relative to the conventional program. Then we describe modifications with optimal cross selection, more than one recurrent selection cycle per year, and lifting the cost constraints.

A cycle of population improvement with truncation selection

We assumed that each cross produced 14 seeds out of which 10 were designated for selection candidates and 4 were designated for production of doubled-haploid lines (passed to the product development component). With 64 crosses there were 640 selection candidates (Table S1.1 and Table 1). We ranked the candidates based on genomic prediction and selected the best 32 or 128 as parents of the next cycle (Table 1), which we respectively denote as TwoPartTS and TwoPartTS+ scenarios; TS stands for truncation selection and + for a larger number of parents. These two scenarios respectively correspond to 5% and 20% selected individuals (selection intensity of 2.06 and 1.40). We use these scenarios to demonstrate the effect of selection and drift caused by the different number of parents. The selected individuals were randomly split into male and female pools to model potential flowering time differences. Assuming that

one wheat plant has four tillers and that we need to produce 64 crosses, we crossed 16 males with 16 females (four crosses per plant) when 32 parents were used or 64 males with 64 females (one cross per plant) when 128 parents were used.

A cycle of product development

The product development component was the same as the conventional program with genomic selection at the headrow stage. The difference was only that both components produced doubled-haploid lines that were evaluated jointly at the headrow stage of the product development component (Fig. 1). This represents a likely application of the two-part program, where a breeder assigns a part of their resources for rapid population improvement and maintains the conventional strategy with specific lines to design specific crosses that improve/combine various properties of these specific lines.

Interactions between the two components

There were three interactions between the population improvement and product development components. First, doubled-haploid lines from the population improvement component were evaluated alongside the product development component lines at the headrow stage. Main results are shown for this joint group of individuals to enable comparison with the conventional programs. Second, genomic predictions in the population improvement component were based on training data collected in product development trials. Third, at the beginning of each year current crossing block lines of the product development component were considered as selection candidates of the population improvement component – in addition to outbred individuals in the population improvement component. We did this to potentially include lines with high genetic merit or increase genetic diversity.

Costs

The two-part program increased costs due to additional genotyping in the population improvement component. We equalized the total cost by decreasing the number of produced doubled-haploid lines (Table S1.1). The number of lines with the two-part program involved lines from the two components.

Optimal cross selection

Application of the optimal cross selection in the population improvement component changed selection of parents and their crossing. The method produced a crossing plan that determined which individuals were selected and how they should be mated to maximise genetic gain at a predefined loss of diversity. Practically this meant that between 32 and 128 individuals contributed to 64 crosses, i.e., a selection candidate could contribute to 0, 1, 2, 3, or 4 crosses. We ran optimal cross selection with a range of penalties on loss of diversity – operationalized with penalty degrees (1°, 5°, 10°, …, 85°; described in the optimal cross selection subsection in the manuscript). We did not use optimal cross selection in the product development component, because we assumed that a breeder would design crosses with specific criteria not controlled by optimal cross selection. However, at the beginning of each year we considered using the latest crossing block lines from the product development part into the optimal cross selection for the population improvement component.

Number of recurrent selection cycles per year

We have evaluated the effect of 1, 2, 3, 4, 5, or 6 cycles of recurrent selection per year assuming that this is possible with the intensive use of greenhouses. Increasing number of cycles per year increases per year genotyping costs. To account for this increase, we have scaled numbers of parents, crosses, and selection candidates per cycle such that the total number of selection candidates per year was approximately constant (640; Table 1).

Lifting cost constraints

Increasing the number of recurrent selection cycles per year increases the number of selection candidates per year and through that genotyping costs. In previous two-part scenarios we have avoided increasing costs by reducing the number of crosses per cycle (Table 1). We have run all these scenario also without cost constraints, i.e., keeping the number of crosses per cycle constant (64) irrespective of the number of cycles per year (Table 1).

References

Chen GK, Marjoram P, Wall JD (2009) Fast and flexible simulation of DNA sequence data. Genome Res 19:136–142 . doi: 10.1101/gr.083634.108

Gaynor RC, Gorjanc G, Bentley AR, et al (2017) A Two-Part Strategy for Using Genomic Selection to Develop Inbred Lines. Crop Sci 57:2372–2386 . doi: 10.2135/cropsci2016.09.0742

Peng JH, Sun D, Nevo E (2011) Domestication evolution, genetics and genomics in wheat. Mol Breed 28:281–301 . doi: 10.1007/s11032-011-9608-4

Thuillet A-C, Bataillon T, Poirier S, et al (2005) Estimation of Long-Term Effective Population Sizes Through the History of Durum Wheat Using Microsatellite Data. Genetics 169:1589–1599 . doi: 10.1534/genetics.104.029553
