# Supplementary material for: Optimal cross selection for long-term genetic gain in two-part programs with rapid recurrent genomic selection
Source: Theor Appl Genet. 2018 Jun 6;131(9):1953–66. doi: 10.1007/s00122-018-3125-3 (PMC6096640; doi:10.1007/s00122-018-3125-3)
Supplement: Supplementary file 2 — Supplementary material 2 (PDF 779 kb) [file 122_2018_3125_MOESM2_ESM.pdf]

Optimal cross selection for long-term genetic gain in a two-part genomic selection strategy

Supplementary Material S2:
Additional results

Gregor Gorjanc*, R. Chris Gaynor, John M. Hickey

G. Gorjanc, R.C. Gaynor, and J.M. Hickey The Roslin Institute and Royal (Dick) School of Veterinary Studies, University of Edinburgh, Easter Bush Research Centre, Midlothian EH25 9RG, UK

*Corresponding author (gregor.gorjanc@roslin.ed.ac.uk)

Fig. S2.1: Genetic mean of doubled-haploid lines (top) and population improvement component (bottom) over 20 years of selection by breeding program and cost constraints; two-part programs used four recurrent selection cycles per year **2**

Fig. S2.2: Genic standard deviation of doubled-haploid lines (top) and population improvement component (bottom) over 20 years of selection by breeding program and cost constraints; two-part programs used four recurrent selection cycles per year **3**

Fig. S2.3: Genetic standard deviation of doubled-haploid lines (top) and population improvement component (bottom) over 20 years of selection by breeding program and cost constraints (mean and 95% confidence interval); two-part programs used four recurrent selection cycles per year **4**

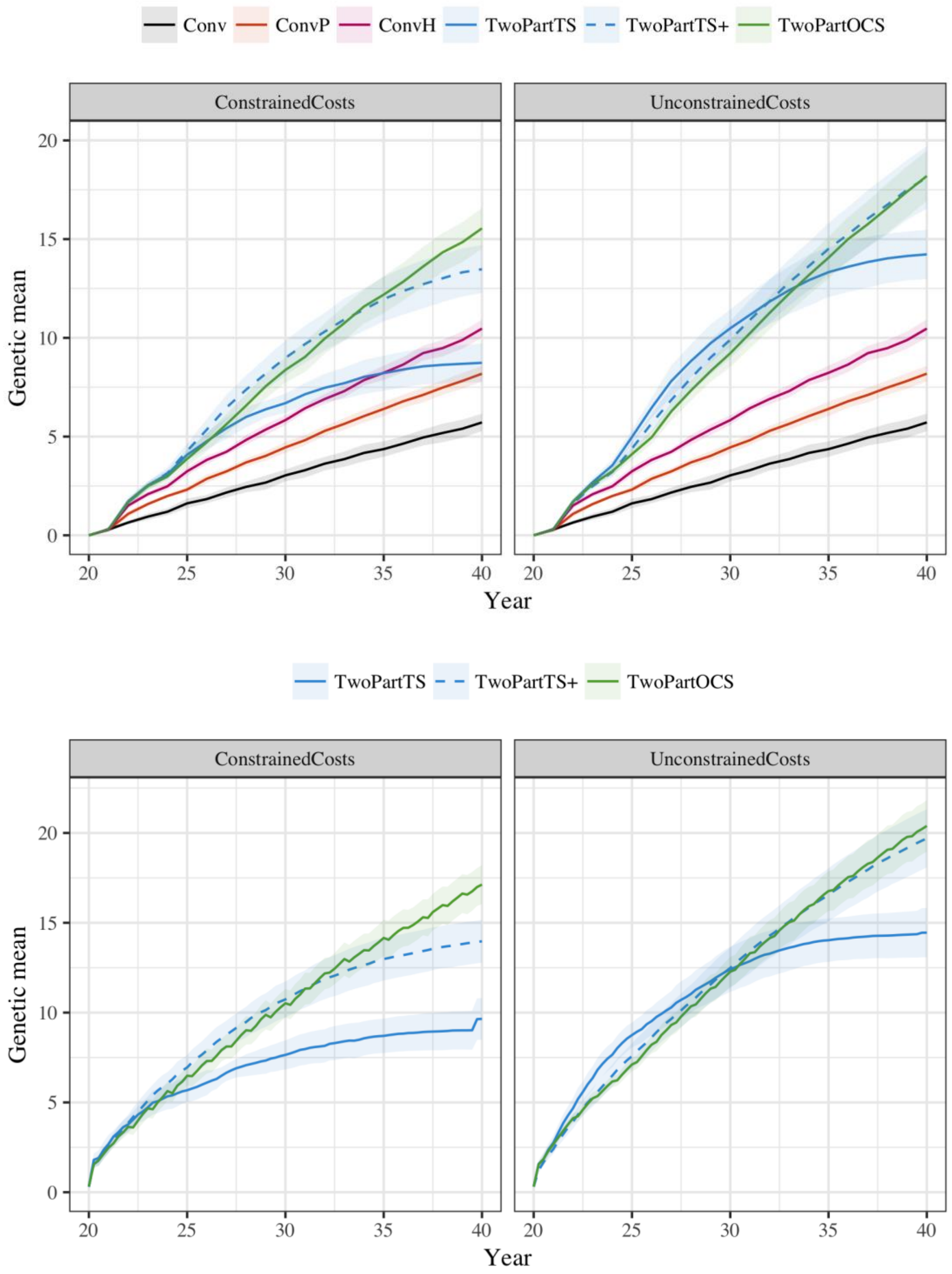

Fig. S2.1: Genetic mean of doubled-haploid lines (top) and population improvement component (bottom) over 20 years of selection by breeding program and cost constraints; two-part programs used four recurrent selection cycles per year

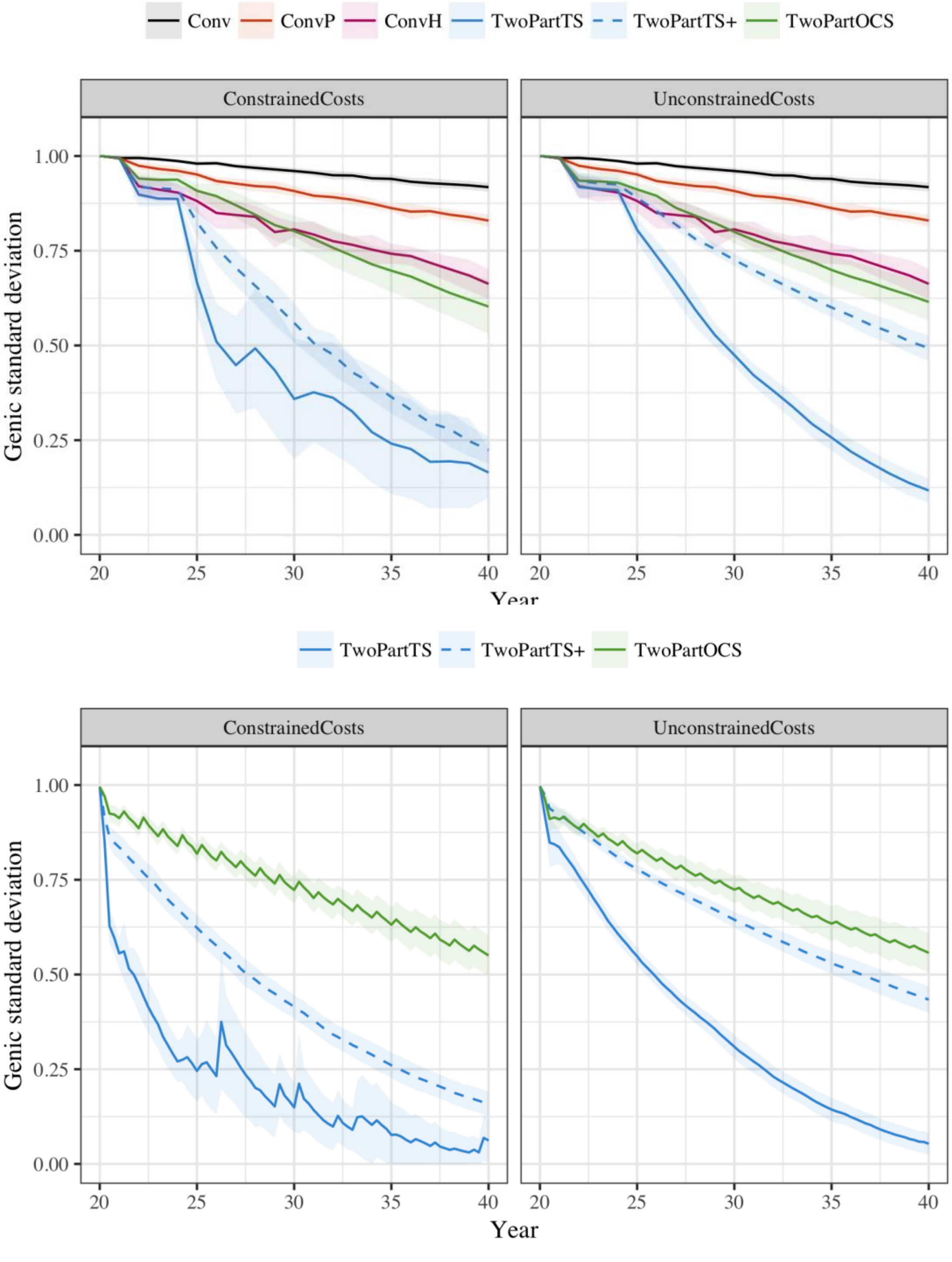

Fig. S2.2: Genic standard deviation of doubled-haploid lines (top) and population improvement component (bottom) over 20 years of selection by breeding program and cost constraints; two-part programs used four recurrent selection cycles per year

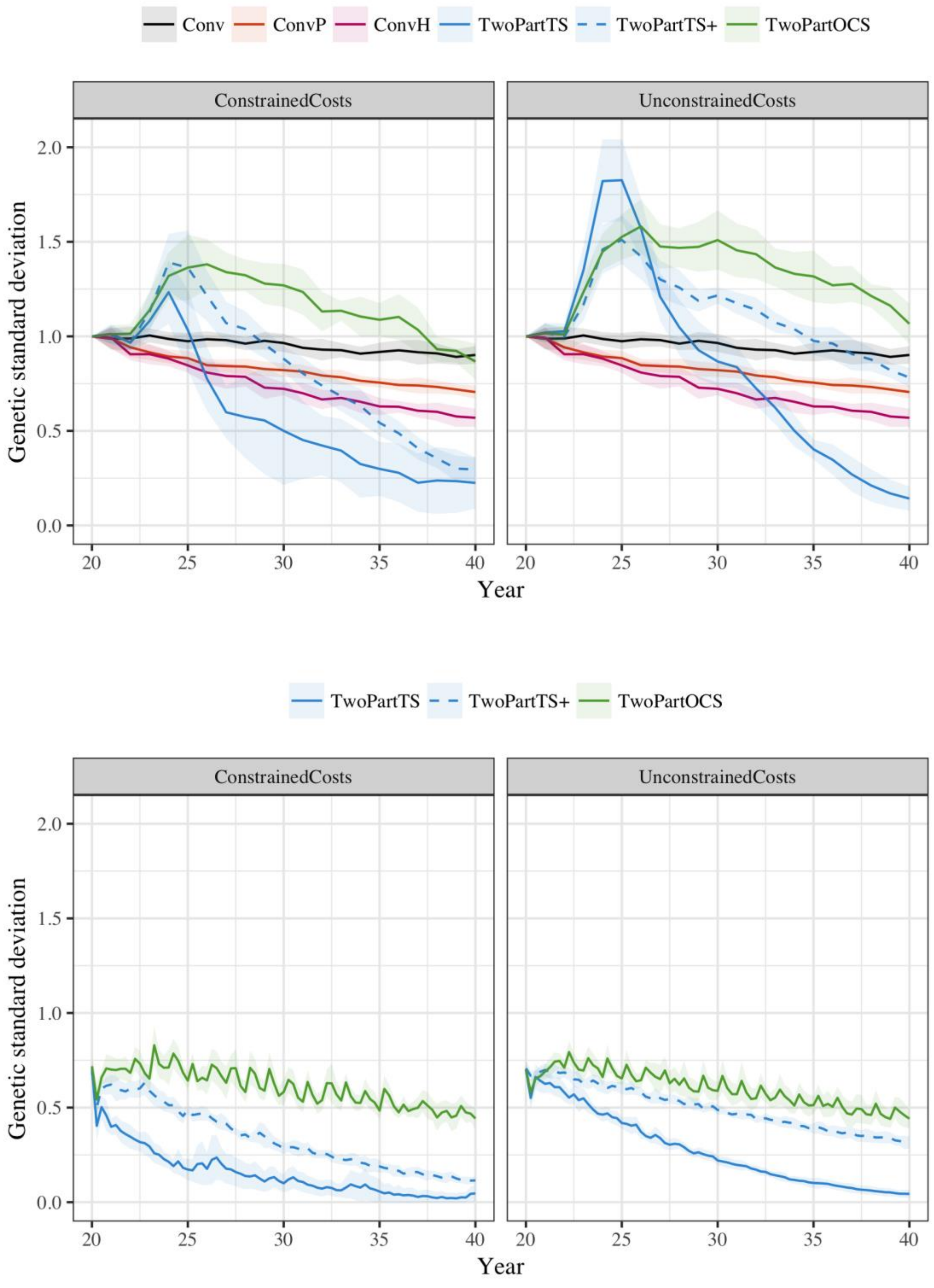

Fig. S2.3: Genetic standard deviation of doubled-haploid lines (top) and population improvement component (bottom) over 20 years of selection by breeding program and cost constraints (mean and 95% confidence interval); two-part programs used four recurrent selection cycles per year
