# Supplementary material for: Optimal cross selection for long-term genetic gain in two-part programs with rapid recurrent genomic selection
Source: Theor Appl Genet. 2018 Jun 6;131(9):1953–66. doi: 10.1007/s00122-018-3125-3 (PMC6096640; doi:10.1007/s00122-018-3125-3)
Supplement: Supplementary file 3 — Supplementary material 3 (PDF 723 kb) [file 122_2018_3125_MOESM3_ESM.pdf]

Optimal cross selection for long-term genetic gain in a two-part genomic selection strategy

Supplementary Material S3: Additional Results with the Cross-Self-Select method

Gregor Gorjanc*, R. Chris Gaynor, John M. Hickey

G. Gorjanc, R.C. Gaynor, and J.M. Hickey The Roslin Institute and Royal (Dick) School of Veterinary Studies, University of Edinburgh, Easter Bush Research Centre, Midlothian EH25 9RG, UK

*Corresponding author (gregor.gorjanc@roslin.ed.ac.uk)

Table S3.1: Breeding program characteristics with the two-part Cross-Self-Select breeding strategy (number of crosses, number of doubled-haploid lines per cross, total number of doubled-haploid lines, and total cost) 2

Table S3.2: Per cycle characteristics of the population improvement component by number of recurrent selection cycles per year with the two-part Cross-Self-Select breeding strategy (number or crosses per cycle, number of selection candidates per cycle, and minimum or maximum number of parents used per cycle) 2

Fig. S3.1: Genetic mean of doubled-haploid lines (top) and population improvement component (bottom) over 20 years of selection by breeding strategy under constrained costs (mean and 95% confidence interval). 3

Fig. S3.2: Genetic standard deviation of doubled-haploid lines (top) and population improvement component (bottom) over 20 years of selection by breeding strategy under constrained costs (mean and 95% confidence interval). 4

Fig. S3.3: Genic standard deviation of doubled-haploid lines (top) and population improvement component (bottom) over 20 years of selection by breeding strategy under constrained costs (mean and 95% confidence interval). 5

We implemented the Cross-Self-Select method by selfing the 640 population improvement candidates. This could generate 50,000 to 80,000 candidate genotypes (= selfing 640 plants with 4 tillers and assuming 20 to 30 seeds per selfed tiller). To equalize costs with other programs (Table S1), we reduced the number of produced doubled-haploid lines per cross (Table S3.1) and generated “only” 10,240 candidates per year (Table S3.2). Out of these candidates we selected 32 or 128 parents with one cycle, which respectively corresponds to 0.31% and 1.25% selected individuals (selection intensity of 3.04 and 2.59). We assumed that the selfing step doubled cycle time. In results below we compare two cycles of the Cross-Self-Select method per year with the four cycles of the Cross-Select method.

Table S3.1: Breeding program characteristics with the two-part Cross-Self-Select breeding strategy (number of crosses, number of doubled-haploid lines per cross, total number of doubled-haploid lines, and total cost)

Strategy	#Crosses	#Lines / cross	#Lines	Cost ($)
TwoPart (Cross-Self-Select)	/	/	2,600	347,800
product development	100	16	1,600	88,000
population improvement	64	15.6	1,000	259,800

Table S3.2: Per cycle characteristics of the population improvement component by number of recurrent selection cycles per year with the two-part Cross-Self-Select breeding strategy (number or crosses per cycle, number of selection candidates per cycle, and minimum or maximum number of parents used per cycle)

			#Parents	
#Cycles	#Crosses	#Candidates	Min	Max
1	64	10,240	32	128
2	32	5,120	16	64
3	22	3,414	12	44

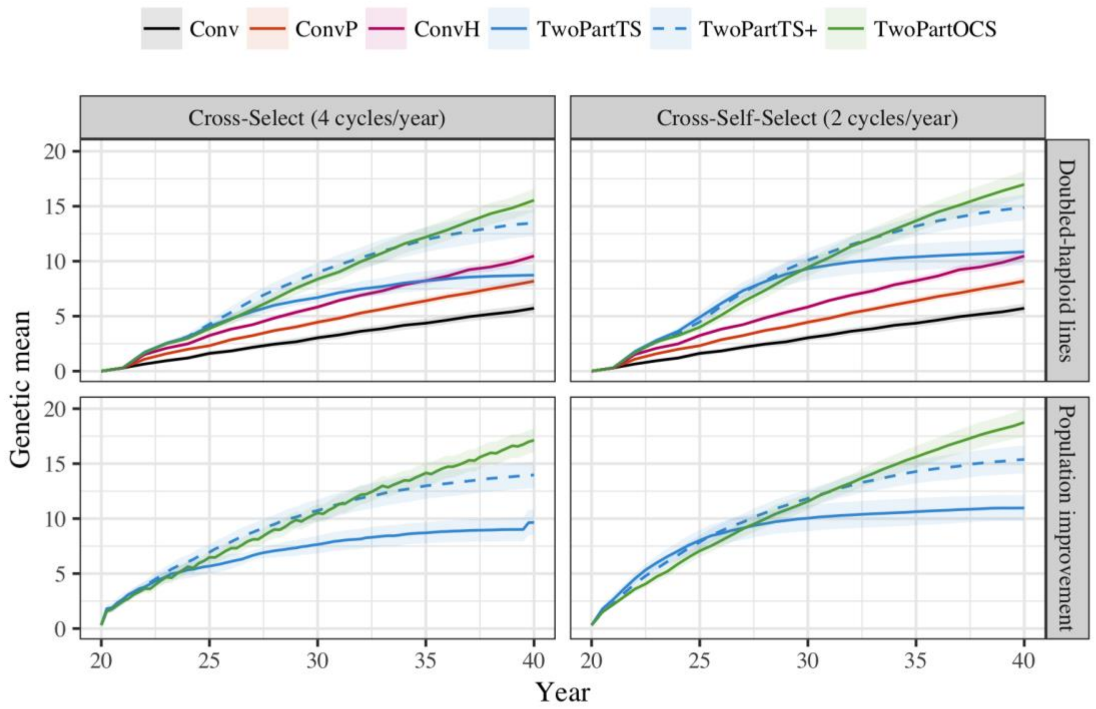

Fig. S3.1: Genetic mean of doubled-haploid lines (top) and population improvement component (bottom) over 20 years of selection by breeding strategy under constrained costs (mean and 95% confidence interval).

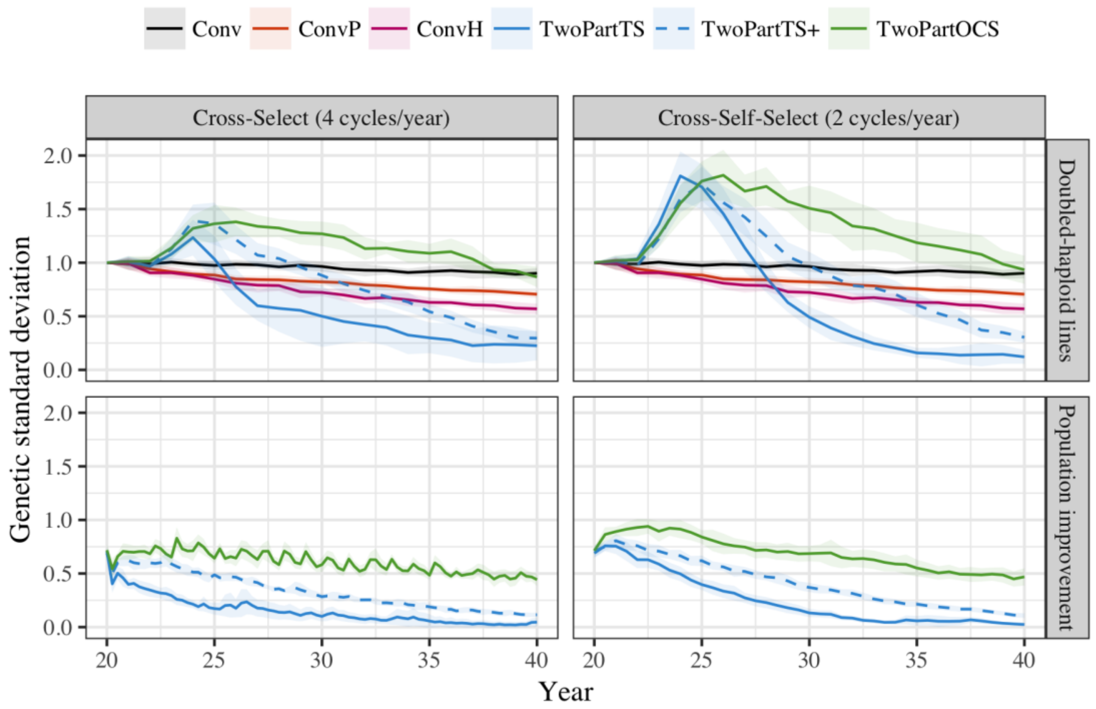

Fig. S3.2: Genetic standard deviation of doubled-haploid lines (top) and population improvement component (bottom) over 20 years of selection by breeding strategy under constrained costs (mean and 95% confidence interval).

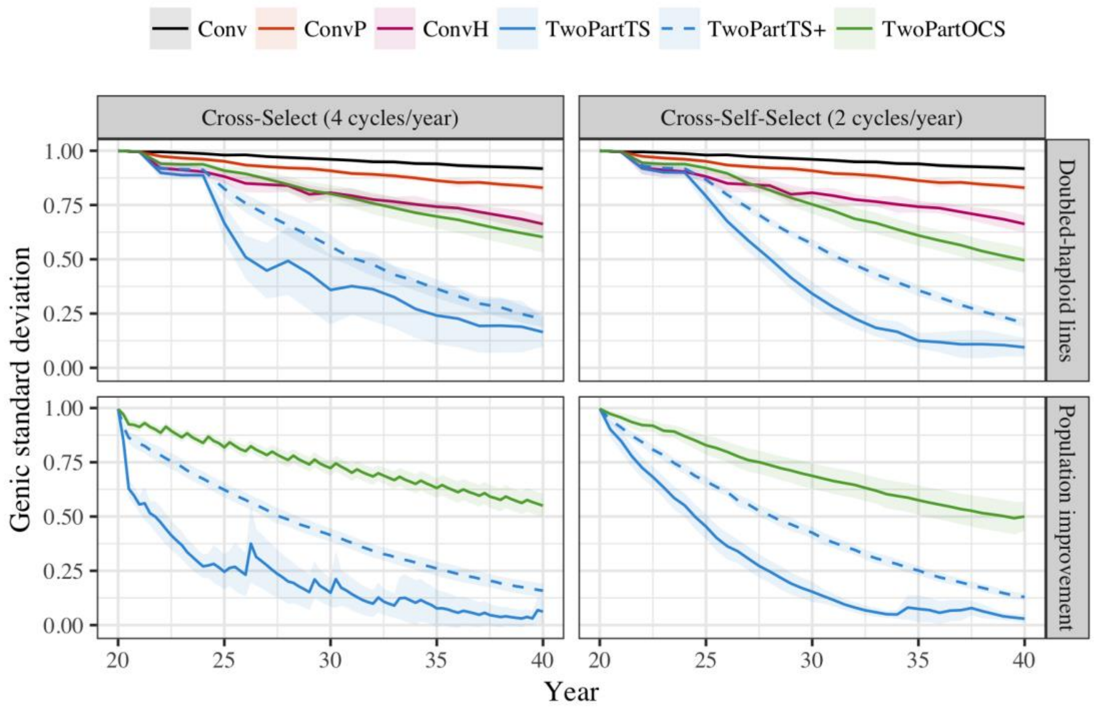

Fig. S3.3: Genic standard deviation of doubled-haploid lines (top) and population improvement component (bottom) over 20 years of selection by breeding strategy under constrained costs (mean and 95% confidence interval).
